# Antioxidant and Anti-Inflammatory Effects of Shungite against Ultraviolet B Irradiation-Induced Skin Damage in Hairless Mice

**DOI:** 10.1155/2017/7340143

**Published:** 2017-08-13

**Authors:** Ma. Easter Joy Sajo, Cheol-Su Kim, Soo-Ki Kim, Kwang Yong Shim, Tae-Young Kang, Kyu-Jae Lee

**Affiliations:** ^1^Department of Environmental Medical Biology, Wonju College of Medicine, Yonsei University, Wonju, Gangwon 220-701, Republic of Korea; ^2^Department of Microbiology, Wonju College of Medicine, Yonsei University, Wonju, Gangwon 26426, Republic of Korea; ^3^Department of Internal Medicine, Wonju College of Medicine, Yonsei University, Wonju, Gangwon 26426, Republic of Korea; ^4^Department of Rheumatology, Wonju College of Medicine, Yonsei University, Wonju, Gangwon 26426, Republic of Korea; ^5^Institute for Poverty Alleviation and International Development (IPAID), Yonsei University, Wonju Campus, Wonju, Gangwon 220-710, Republic of Korea

## Abstract

As fullerene-based compound applications have been rapidly increasing in the health industry, the need of biomedical research is urgently in demand. While shungite is regarded as a natural source of fullerene, it remains poorly documented. Here, we explored the *in vivo* effects of shungite against ultraviolet B- (UVB-) induced skin damage by investigating the physiological skin parameters, immune-redox profiling, and oxidative stress molecular signaling. Toward this, mice were UVB-irradiated with 0.75 mW/cm^2^ for two consecutive days. Consecutively, shungite was topically applied on the dorsal side of the mice for 7 days. First, we found significant improvements in the skin parameters of the shungite-treated groups revealed by the reduction in roughness, pigmentation, and wrinkle measurement. Second, the immunokine profiling in mouse serum and skin lysates showed a reduction in the proinflammatory response in the shungite-treated groups. Accordingly, the redox profile of shungite-treated groups showed counterbalance of ROS/RNS and superoxide levels in serum and skin lysates. Last, we have confirmed the involvement of Nrf2- and MAPK-mediated oxidative stress pathways in the antioxidant mechanism of shungite. Collectively, the results clearly show that shungite has an antioxidant and anti-inflammatory action against UVB-induced skin damage in hairless mice.

## 1. Introduction

Ultraviolet (UV) radiation often causes various skin diseases [1]. At short-term UV exposure, it could suppress immune function, and at chronic exposure, it could lead to photoaging and/or carcinoma [2, 3]. Skin damage induced by UV irradiation includes photosensitivity, erythema, and DNA damage resulting in invisible changes of cell and gene level [4–6]. These involve alterations in immune response such as increased mast cells, outburst of cytokines by keratinocytes, and suppressed levels of Langerhans cells [7–9]. It is generally known that the skin is the first line of defense in our immune system that causes it to be one of the primary candidates and targets of oxidative stress [10, 11]. Reactive oxygen species (ROS) and reactive nitrogen species (RNS) have a major participation in the pathogenesis of UV-induced skin damage by both direct DNA damage and indirect ROS-mediated oxidative damage [12–14]. Furthermore, the immune dysfunction and ROS would aggravate the skin barrier structure and function, consequently leading to photoaging [15]. With these reports, targeting ROS-induced cellular damage or immune dysfunction in skin barrier is a strategic move to prevent UV-induced skin damage. A plethora of antioxidant agents in different forms are readily available such as cosmetics, inhalant, and foods to reduce UV-induced skin damage [16–20]. However, convenient treatments or manipulations of UV-induced skin injury are still limited.

According to scholars, even if shungite has been around for billions of years, the present comprehension of this promising mineral is underway. Scientists have shown interest in the study of carbon from shungite rocks for over two centuries focusing mainly in the structural, chemical, and geological investigations [21]. Shungite rocks contain one of the oldest noncrystal carbon found to be originated in a village named Shunga in the Karelian shore of the Lake Onega (Russia) [21]. The carbon in shungite rocks penetrates in nearly all rocks of the region over an area in excess of 9000 km^2^ [21]. Shungite rocks are divided into five types based on the carbon percentage (1–98 wt.%) [22]. Type I is the rocks that occur in shungite deposits containing the highest amount of carbon (96–98 wt.%) with traces of other elements (0.1–0.5% H, 0.6–1.5% O, 0.7–1.0% N, and 0.2–0.4% S) [23]. On the other hand, type III is the most common one with 30 wt.% of carbon. In the ancient times, shungite is said to be related to allergies, skin diseases, diabetes mellitus, stomatitis, periodontal disease, hair loss, cosmetic flaws, and many other diseases [21]. It is characterized by high reactivity in thermal processes, high absorption, catalytic properties, electrical conductivity, and chemical stability. Shungite particles, regardless of their size, have bipolar properties [21]. By the end of the twentieth century, scientists had partially explained the reasons for the beneficial effect of shungite. As it turned out, this mineral is mainly composed of carbon, much of which is represented by the spherical molecules of fullerenes. Eventually, the revelation of fullerene in shungite rocks provided a new impetus to the exploration of shungite [24].

In this study, we explored the therapeutic *in vivo* effects of shungite against UVB-induced skin damage comparing the antioxidant power of mineral-rich shungite and mineral-less shungite with commercially available fullerene C60. We hypothesize that shungite might have possible antioxidant and anti-inflammatory effects against ultraviolet B- (UVB-) induced skin damage in hairless mice. To verify this hypothesis, we investigated the physiological skin parameters, immune-redox profiling, and oxidative stress-related signaling of mineral-rich and mineral-less shungite-treated hairless mice.

## 2. Materials and Methods

### 2.1. Mice

Eight-week-old male hairless mice were obtained from Orient Bio Inc. (Seongnam, Republic of Korea) and were maintained at 22 ± 2°C and 40–60% humidity under a cycle of 12 : 12 hr light dark. The mice were acclimatized for one week and randomly assigned into six groups: the non-UVB-irradiated normal control group (NC), and the five UVB-irradiated groups: no treatment group (UV), fullerene-treated group (PC), olive oil-treated group (OIL), mineral-rich shungite-treated group (MRS), and mineral-less shungite-treated group (MLS). About 200 *μ*L of treatment compounds (200 *μ*g/mL) was topically administered using the hands with gloves applying the same pressure all throughout the dorsal side of the mouse. At the end of the experiment, mice were euthanized through inhalant anesthetics, CO_2_ gas for 20 seconds, and then the mice were decapitated. The animal use and protocol for this experiment was approved by the Institutional Animal Care and Use Committee (IACUC), Yonsei University Wonju Campus (YWC-151113-1).

### 2.2. UVB Exposure

The experimental mice were placed in a plastic cage and were irradiated using TL 40 W/12RS Philips, an unfiltered fluorescent sun lamps (Amsterdam, Netherlands) emitting rays of 290–320 nm. The distance from the lamps to the dorsal skin surface of the mice was 20 cm. The intensity of UVB lamp was 0.75 ± 0.10 mW/cm^2^ using YK-34 UV, a UV light meter by Lutron Electronics Inc. (Taipei, Taiwan), and the total cumulative dose energy of UVB irradiated was 2700 mJ/cm^2^. The mice were irradiated for 15 mins in 2 consecutive days.

### 2.3. Chemical and Shungite Suspension Preparation

Natural shungite stone was purchased from Karelian Shungite Factory (Republic of Karelia, Russia). Natural shungite has 2.3–2.4 g/cm^3^ of specific gravity, 0.5% of porosity, 1000–1500 kgf/cm^2^ of compressed strength, and 1100–1200 kg/m^3^ of density and is composed of 28% carbon, 57% SiO_2_, 4.3% Al_2_O_3_, 2.8% FeO, 1.5% K_2_O, 1.5% S, 1.2% MgO, 0.3% CaO, 0.2% TiO_2_, 0.2% Na_2_O, and 3% H_2_O crystal. Mineral-rich shungite (MRS) and mineral-less shungite (MLS) were produced by MST Technology Ltd. (Incheon, Republic of Korea) through the treatment processes such as grinding of stone, treatment of alkaline solution and acidic solution, washing and filtration, treatment of boron (B), and high-temperature treatment process. The property and components of shungite vary according to the pulverized particle size of stone, mixing proportion of alkaline solution, acidic solution, and boron-containing compound with shungite powder, concentration of treatment solutions, and heating time and temperature. MRS and MLS powder used as an experimental material were refined to increase carbon ratio (MRS: 86.4%, NLS: 99.4% by energy dispersive X-ray spectroscopy (Missouri, USA)) using different treatment processes, and fullerene-like carbons such as C55, C60, C70, C74, C93, and C112 were mainly detected by MALDI-TOF mass spectrometry (Shimadzu Corp., Kyoto, Japan). Carbon powder were mixed with olive oil from Sigma-Aldrich Co. LLC. (Darmstadt, Germany) as a vehicle for the topical treatment on the skin.

Manufactured buckminsterfullerene C60 was purchased from Vaughter Wellness (London, United Kingdom) with 99.8% purity. Mineral-rich shungite was treated with KOH as an alkaline treatment with high temperature. Mineral-less shungite was treated with HNO_3_ and high-temperature (3000°C) treatment of mineral-rich shungite. These mixtures were sonicated using a vortex and sonicator until all of the solutions were mixed vigorously.

### 2.4. Characterization of Mineral-Less Shungite

Composition and visualization of shungite with mineral-less were analyzed by Energy-dispersive X-ray (EDX) spectroscopy. The mineral percent composition includes 86.43% carbon, 0.18% sodium, 1.33% magnesium, 3.17% silicon, 1.09% sulfur, 0.22% chlorine, 0.95% potassium, 5.33% calcium, 1.06% iron, and 0.2% copper.

### 2.5. Physiological Analysis of the Skin Surface

Skin condition of the mice was assessed before and after 7-day treatment with a device for skin diagnostics, Aramo TS Device (Seongnam, Republic of Korea). The device is a comprehensive system for noninvasive, optical analysis of several dimensionless parameters of the skin: moisture, elasticity, sebum porosity, smoothness (evenness), discolourations (pigmentation), and wrinkles. The measurements were taken in very specific points located on the dorsal side of the mice, and the results were the difference before and after treatment to assess the improvement.

### 2.6. Immune Profile

#### 2.6.1. White Blood Cell (WBC) and Its Differential Count Analysis

Blood was collected from the retro-orbital plexus in tubes coated with anticoagulant and was mixed with an automatic mixer for 5 min. Thereafter, WBC and its differential members such as lymphocytes, monocytes, neutrophil, eosinophil, and basophil were measured using an automatic blood analyser by HEMAVET HV950 FS, Drew Scientific Inc. (Texas, USA).

#### 2.6.2. Serum and Skin Lysate Inflammatory Cytokines

Serum and skin lysate inflammatory cytokines including IL-1*β*, IL-6, IL-10, IL-17, IL-KC, and TNF-*α* were analyzed using Bio-Plex Cytokine Assay by Bio-Rad (California, USA) according to the manufacturer's instructions. Standard curves for each adipokine and cytokine were generated by using the reference concentrations provided in the kits. Mean fluorescence intensity was acquired on a Luminex technology by Bio-Rad's Bio-Plex 200 system Multiplex Bead Array System™ (California, USA) and analyzed with associated software using a 5-parameter logistic method.

### 2.7. Redox Profile

#### 2.7.1. Intracellular Reactive Oxygen Species (ROS) Detection

ROS/RNS detection kit by Enzo Life Sciences Inc. (New York, USA) was used according to the manufacturer's instructions to determine effects of shungite compounds on oxidative stress and superoxide in serum and skin lysates. Twenty-five (25) *μ*L sample was loaded, and detection solution was added to each well. The plate was read in the DTX-800 multimode microplate reader by Beckman Counter Inc. (California, USA) using a filter set of 485/20 excitation and 528/20 emission to detect oxidative species.

#### 2.7.2. NO Assay

The nitrite (NO_2_^−^) present in the blood serum and skin lysates of mice was detected using the Griess reagent by Promega Corp. (Madison, USA). Briefly, 50 *μ*L of the serum was mixed with an equal volume of Griess reagent in a 96-well microtiter plate and incubated at room temperature for 15 min. The absorbance was read at 540 nm using a DTX-880 multimode microplate reader by Beckman Counter Inc. (California, USA). The NO_2_^−^ concentration was calculated by comparison with the representative NO_2_^−^ standard curve generated by serial two-fold dilutions of sodium nitrate.

#### 2.7.3. Antioxidant Endogenous Enzyme Activities

The activity of superoxide dismutase (SOD), glutathione peroxidase (GPx), and myeloperoxidase (MPO) in serum and skin lysates was measured using the Biovision kit (California, USA). The crude skin lysate was centrifuged at 14,000 rpm for 15 min. at 4°C, and the debris was discarded. Skin lysate was then checked for protein concentration using Pierce™ BCA Protein Assay Kit by Thermo Scientific (Illinois, USA). Finally, the normalized concentrations of cell lysates were used to measure the activities of different antioxidant enzymes (SOD, GPx, and MPO) according to the manufacturer's instruction.

### 2.8. Western Blot Analysis

Briefly, the prepared skin lysate with the normalized protein concentration was equally loaded and separated by electrophoresis on SDS-polyacrylamide gels. Ten (10) percent separating gel and 5% stacking gel were prepared with the following components. 10% separating gel (10 mL) consisted of 4.0 mL dH_2_O, 3.3 mL 30% acrylamide mix, 2.5 mL 1.5 M Tris (pH 8.8), 100 *μ*L 10% SDS, 100 *μ*L 10% APS, and 4 *μ*L TEMED. 4% stacking gel (5 mL) consist of 2.7 mL dH_2_O, 670 *μ*L 30% acrylamide mix, 500 *μ*L 1.0 M Tris (pH 6.8), 40 *μ*L 10% SDS, 40 *μ*L 10% APS, and 4 *μ*L TEMED. The 15 *μ*L ~ 20 *μ*L of the sample with sample loading buffer with optimized concentration was loaded in the gel. Then, it will be run in 30 mA, 70–80 v. The PVDF membranes were blocked with 5% nonfat skim milk at room temperature for 2 hr and were incubated with the following primary antibodies: phospho-JNK and phospho-p38, ERK, Nrf2, and *β*-actin (dilution: 1 : 2000; Cell Signaling Technology, Massachusetts, USA) in Tris-buffered saline/tween 20 (1X TBST) containing 5% bovine serum albumin overnight in 4°C. The secondary antibody used was anti-rabbit (dilution: 1 : 2000; Cell Signaling Technology), and then it was incubated at room temperature for 2 hr. Specific protein bands were visualized by the enhanced chemiluminescence (ECL Pierce Biotechnology) using UVP Biospectrum 600 Imaging System (UVP, LLC, Upland, CA, USA). *β*-Actin (dilution: 1 : 2000, Cell Signaling Technology) was used as loading controls for the total protein content.

### 2.9. Data Management and Analysis

Statistical analysis was carried out using analysis of variance (ANOVA) followed by subsequent multiple comparison test (Dunnett's multiple comparison test) using GraphPad Prism version 7.0 software packages (California, USA). Differences were considered significant at ^∗^*p* < 0.05, ^∗∗^*p* < 0.01, ^∗∗∗^*p* < 0.001, and ^∗∗∗∗^*p* < 0.0001.

## 3. Results and Discussion

### 3.1. The Effects of Shungite on the Physiological Skin Surface of the Mice

This study aims to examine whether topical application of MRS and MLS could attenuate the related skin damage caused by UVB irradiation in hairless mice compared to the pure fullerene C60 positive control. We also compared the experimental groups (MRS and MLS groups) to non-UVB-irradiated group, negative control UVB-irradiated only group, and UVB + olive oil-treated group. We used two experimental groups (MRS is close to the natural shungite that has the most mineral present while the MLS is with least mineral material) to explore the effects of the two different types and also to rule out the effects of other minerals present in the material. On the other note, olive oil was used in the experiment because it has been used as a solubilizing excipient or vehicle in our positive control fullerene C60. Studies have shown that olive oil is the most biocompatible organic solvent for C60 fullerene [25]. Olive oil has been used as an alternative for the toxic glycerol or toluene. In addition, olive oil is one of the most effective and safe solubilizing excipients easily available [26]. However, since olive oil itself is already known to have its antioxidant effects and was also found to have protective effect after UVB exposure [27], it is essential to compare its effect with the olive oil only treatment with the experimental groups which used olive oil as a vehicle.

**Figure 1:**
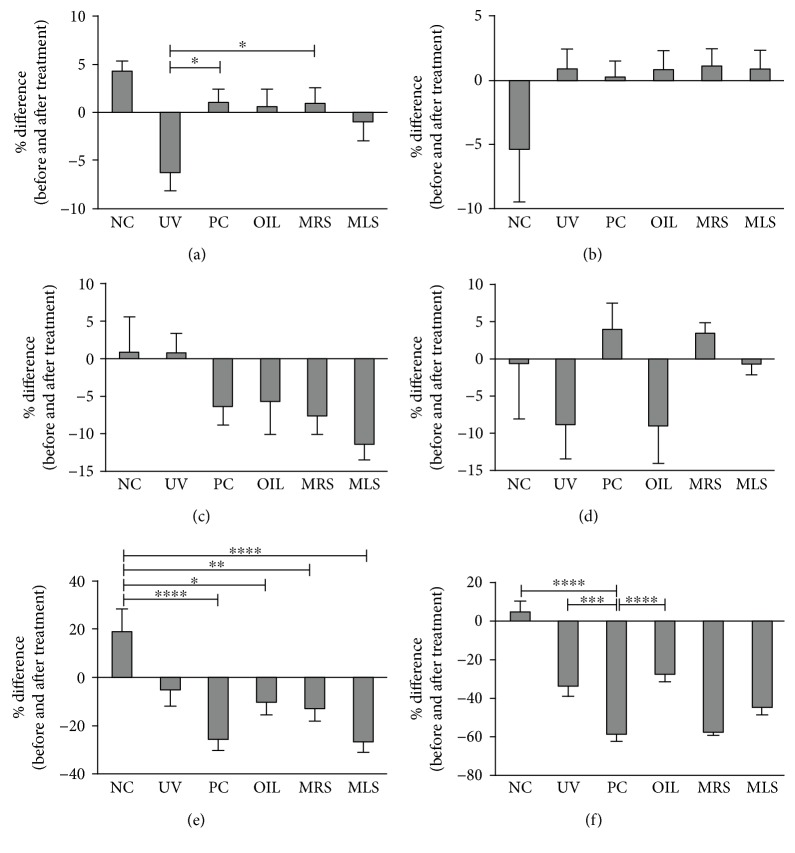
Mineral-rich and mineral-less shungite-treated groups improved physiological skin parameters. Shown are the effects of mineral-rich shungite (MRS) and mineral-less shungite (MLS) on the mouse skin moisture (a), elasticity (b), roughness (c), pore size (d), pigmentation (e), and wrinkle (f). Control groups include non-UVB-irradiated group (NC), UVB-irradiated only group (UV), UVB + fullerene C60-treated positive control group (PC), and UVB + olive oil-treated group (OIL), while experimental groups include UVB + mineral-rich shungite-treated group (MRS) and mineral-less shungite-treated group (MLS). Statistical analysis was carried out using analysis of variance (ANOVA) followed by subsequent multiple comparison test (Tukey's multiple comparisons test) using GraphPad Prism version 5.0. Values are mean ± SD, *n* = 9. Differences were considered significant at ^∗^*p* < 0.05, ^∗∗^*p* < 0.01, ^∗∗∗^*p* < 0.001, and ^∗∗∗∗^*p* < 0.0001.

To verify the clinical evaluation of the skin, we checked different skin parameters and we found that the skin moisture and elasticity of the shungite-treated (MRS and MLS) mice had improved compared to the NC and UV groups, comparable to PC (Figure 1). Furthermore, the roughness, pigmentation, and wrinkle had been significantly reduced and showed improvement after 7 days of topical application of shungite. Pore size of MRS and MLS groups did not show significant reduction, but it has improved compared to the PC. These results show clinical evidences of the antioxidant and anti-inflammatory effects of MRS and MLS in the outward skin surface.

### 3.2. Immune Profiling

#### 3.2.1. Total WBC and Its Differential Count

Total WBC counts were used to evaluate the overview of the severity of inflammation response.  Table 1 showed the total WBC count suppression of MRS and MLS compared to UV and PC. The altered WBC counts may be indicative of direct or indirect effects of the compound treatment on cellular toxicity and proliferation. The recruitment of total WBC and differential counts are highest in PC followed by MLS then MRS. Overall, the suppressed count of the total WBC in the negative control group (UV) was rescued by the shungite-treated groups as well as their differential counts on both MRS and MLS groups after 7 days of topical application of shungite.

#### 3.2.2. Proinflammatory Cytokine Levels in Serum and Skin Lysates of Shungite-Treated UVB-Induced Mice

**Figure 2:**
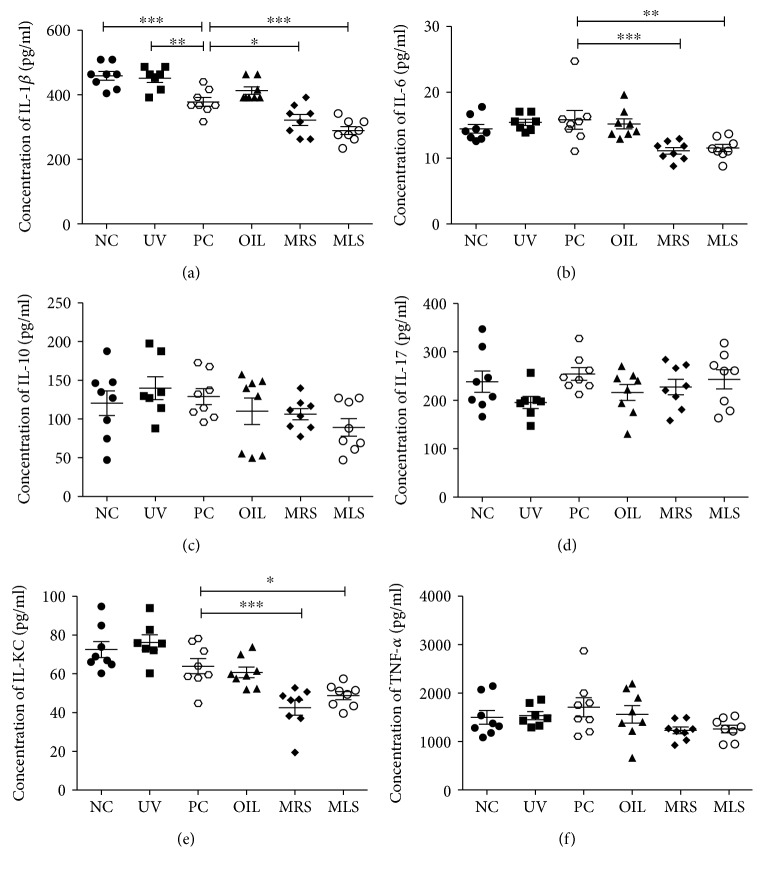
Serum proinflammatory cytokine levels in mineral-rich and mineral-less shungite groups showed immunomodulatory effect. Shown are the serum cytokine concentrations of IL-1*β* (a), IL-6 (b), IL-10 (c), IL-17 (d), IL-KC (e), and TNF-*α* (f) determined using multiplex assay in shungite-treated UVB-induced mice. Control groups include non-UVB-irradiated group (NC), UVB-irradiated only group (UV), UVB + positive control fullerene C60-treated group (PC), and UVB + olive oil-treated group (OIL), while experimental groups include UVB + mineral-rich shungite-treated group (MRS) and mineral-less shungite-treated group (MLS). Statistical analysis was carried out using analysis of variance (ANOVA) followed by subsequent multiple comparison test (Tukey's multiple comparisons test) using GraphPad Prism version 5.0. All values are represented as mean ± SD (*n* = 9), ^∗^*p* < 0.05, ^∗∗^*p* < 0.01, and ^∗∗∗^*p* < 0.001 versus each treatment.

**Figure 3:**
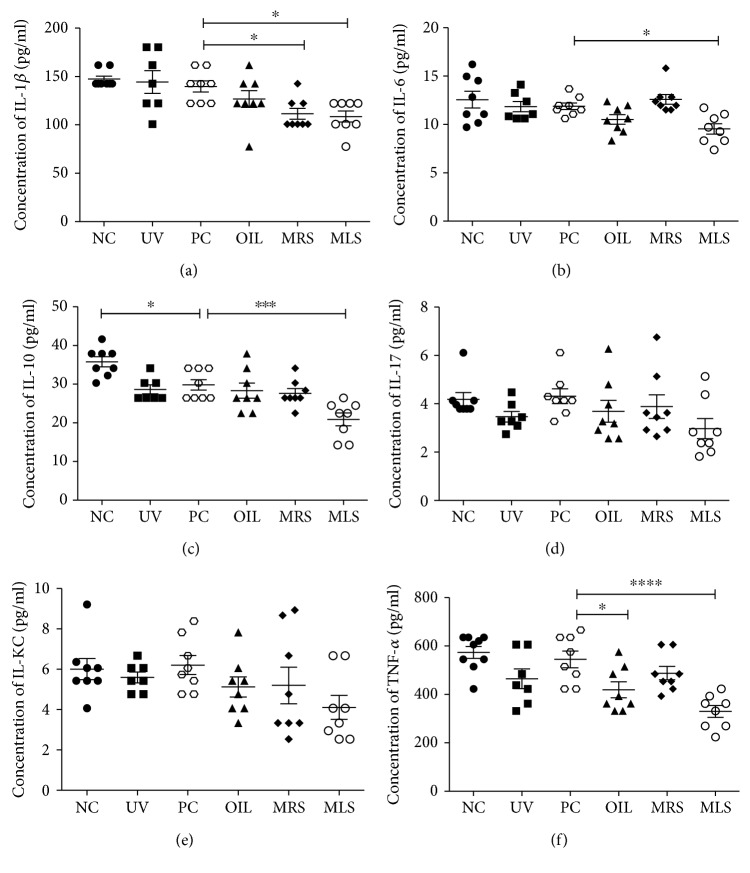
Skin lysate proinflammatory cytokine levels in mineral-rich shungite (MRS) and mineral-less shungite (MLS) groups showed immunomodulatory effect. Shown are the skin lysate cytokine concentrations of IL-1*β* (a), IL-6 (b), IL-10 (c), IL-17 (d), IL-KC (e), and TNF-*α* (f) determined using multiplex assay in shungite-treated UVB-induced mice. Control groups include non-UVB-irradiated group (NC), UVB-irradiated only group (UV), UVB + positive control fullerene C60-treated group (PC), and UVB + olive oil-treated group (OIL), while experimental groups include UVB + mineral-rich shungite-treated group (MRS) and mineral-less shungite-treated group (MLS). Statistical analysis was carried out using analysis of variance (ANOVA) followed by subsequent multiple comparison test (Tukey's multiple comparisons test) using GraphPad Prism version 5.0. All values are represented as mean ± SD, *n* = 9, ^∗^*p* < 0.05, ^∗∗∗^*p* < 0.001, and ^∗∗∗∗^*p* < 0.0001 versus each treatment.

Further evidence showed that shungite might mediate inflammation response that can influence immunological homeostasis. To delineate this proposition, we measured the serum (Figure 2) and skin lysate (Figure 3) cytokine profiling to gauge the inflammatory cytokine balance. The overall trend of the serum levels of IL-1*β*, IL-6, IL-10, IL-KC, and TNF-*α* concentration in shungite-treated mice was lower than that of all the control groups except for IL-17. The UV group showed slightly higher than the NC group. Comparing cytokine levels of serum, MLS showed lower level of proinflammatory cytokines (IL-1*β*, IL-6, and TNF-*α*) as well as the anti-inflammatory cytokine (IL-10) than the PC group (Figure 2). Similar trend was observed in skin lysate levels of IL-1*β*, IL-6, IL-10, IL-KC, and TNF-*α*, except IL-17 concentrations in shungite-treated (Figure 3). In shungite-treated group, lower level of proinflammatory cytokines (IL-1*β*, TNF-*α*, and IL-6) and regulatory cytokines in skin keratinocytes such as IL-17 and IL-KC is highly compatible with the redox profiling results. Cumulative evidence showed that UVB leads to a shift from the production of proinflammatory cytokines such as IL-1*β*, IL-12, IL-8, TNF-*α*, and IFN-*γ* to the production of anti-inflammatory cytokines such as IL-4, IL-10, and IL-13 [28, 29]. Breakdown of pro- and anti-inflammatory cytokines leads to various diseases such as atopic dermatitis, rheumatoid arthritis, and psoriasis. As observed in the results, a similar trend was seen in shungite-treated group in IL-1*β* and TNF-*α*. This may be ascribed to the synergism between two cytokines. On the other hand, the role of IL-6 in UVB-induced skin immune responses is to regulate the release and production of other proinflammatory cytokines [30]. The mechanism which is regulated by IL-17 was not noticeable in our results. IL-KC appears to have the key role in immunosuppressive effects of UVB as it mediates IL-10 which is known to be an anti-inflammatory cytokine [31]. Based on cytokine profiling, shungite treatment restored the inflammatory cytokine imbalance evoked by UV irradiation. UVB irradiation on the skin would induce alteration of antigen-presenting cells including Langerhans cells and imbalance of a cell-mediated immune response. IL-10 is known as an important immunoregulatory cytokine to downregulate inflammatory responses and regulate differentiation and proliferation of several immune cells such as T cells, B cell, natural killer (NK) cells, mast cells, and granulocytes [32]. In that context, this immunomodulation of shungite might justify the antioxidant effect and potential clinical therapeutic usage for skin and oxidative stress disorders [33].

### 3.3. Redox Profiling

#### 3.3.1. Effects of Shungite on the Levels of Total Intracellular ROS and Nitric Oxide (NO) in Mouse Serum and Skin

**Figure 4:**
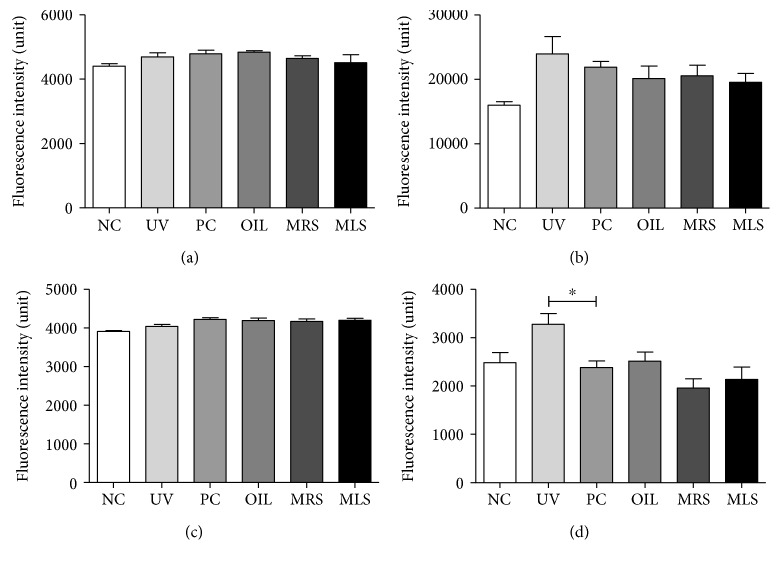
Reduction of total intracellular reactive oxygen/nitrogen (ROS/RNS) and superoxide dismutase (SOD) species in mouse serum and skin lysate of shungite-treated groups. Shown are the effects of shungite in the total ROS/RNS in serum (a) and skin lysates (b) as well as the SOD concentration in serum (c) and skin lysates (d) as determined by an ROS/RNS detection kit by Enzo Life Sciences Inc. Control groups include non-UVB-irradiated group (NC), UVB-irradiated only group (UV), UVB + fullerene C60-treated positive control group (PC), and UVB + olive oil-treated group (OIL), while experimental groups include UVB + mineral-rich shungite-treated group (MRS) and mineral-less shungite-treated group (MLS). Statistical analysis was carried out using analysis of variance (ANOVA) followed by subsequent multiple comparison test (Tukey's multiple comparisons test) using GraphPad Prism version 5.0. Data were expressed as mean fluorescence ± SD, *n* = 9. Differences were considered significant at ^∗^*p* < 0.05 versus each treatment.

**Figure 5:**
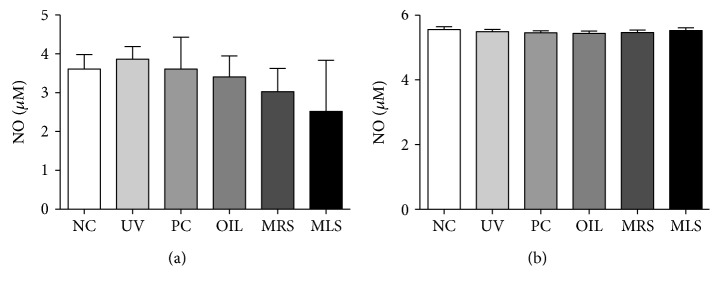
Slight increase of nitric oxide (NO) levels in mouse serum and skin lysates of shungite-treated groups. Shown are the NO levels in serum (a) and skin lysates (b) that were determined using Greiss reagent. Control groups include non-UVB-irradiated group (NC), UVB-irradiated only group (UV), UVB + fullerene C60-treated positive control group (PC), and UVB + olive oil-treated group (OIL), while experimental groups include UVB + mineral-rich shungite-treated group (MRS) and mineral-less shungite-treated group (MLS). Statistical analysis was carried out using analysis of variance (ANOVA) followed by subsequent multiple comparison test (Tukey's multiple comparisons test) using GraphPad Prism version 5.0. Data were expressed as mean ± SD, *n* = 9.

The generation of intracellular ROS and superoxide of both skin lysates in MRS and MLS had reduced levels compared to the high levels of ROS in UV group. However, the reduction of ROS/RNS was not that dramatical in the circulating serum (Figure 4). On the other hand, the NO levels in serum and skin lysates in shungite-treated groups were not significant but a lower trend had been observed in the serum NO levels in shungite-treated group (Figure 5). NO plays a pivotal role in macrophage-mediated cytotoxicity by acting as an effector molecule. NO is a reactive molecule that reacts with ROS to produce reactive nitrogen species; moreover, it is recognized as a mediator and regulator of immune responses. NO has various physiological and pathophysiological responses depending on its relative concentration.

#### 3.3.2. Serum and Skin Lysate Scavenging Enzyme Activity

**Figure 6:**
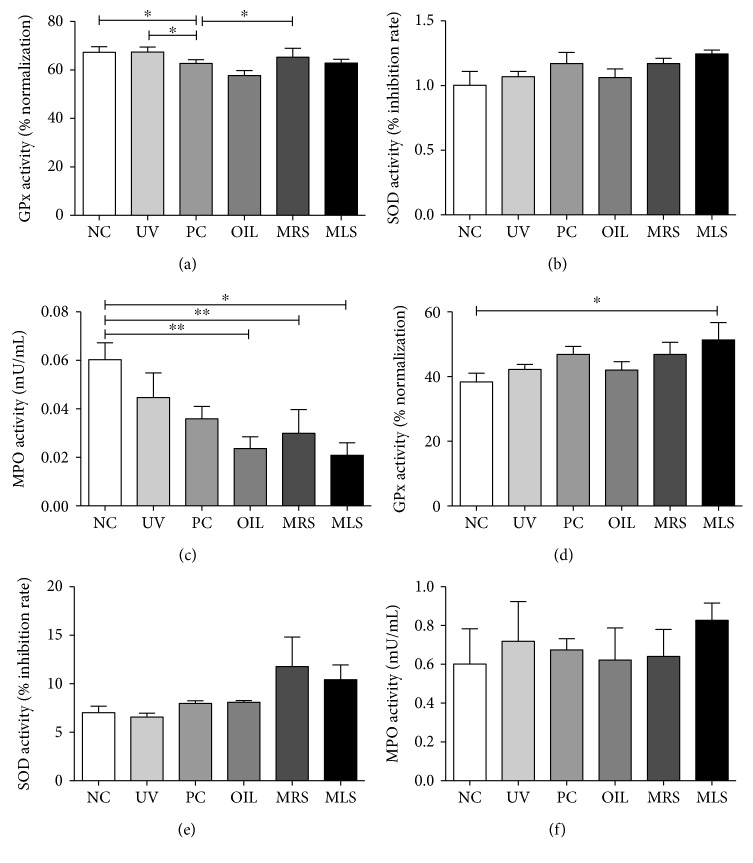
Antioxidant enzyme activities of serum and skin lysates in shungite-treated mice groups: glutathione peroxidase (GPx), superoxide dismutase (SOD), and myeloperoxidase (MPO) levels. Shown are the antioxidant biological enzyme activities such GPX in serum (a) and skin lysates (d), SOD in serum (b) and skin lysates (e), and MPO in serum (c) and skin lysates (f) of shungite-treated UVB-induced mice. Control groups include non-UVB-irradiated group (NC), UVB-irradiated only group (UV), UVB + fullerene-treated positive control group (PC), and UVB + olive oil-treated group (OIL), while experimental groups include UVB + mineral-rich shungite-treated group (MRS) and mineral-less shungite-treated group (MLS). Statistical analysis was carried out using analysis of variance (ANOVA) followed by subsequent multiple comparison test (Tukey's multiple comparisons test) using GraphPad Prism version 5.0. Data were expressed as mean ± SD, *n* = 9, ^∗^*p* < 0.05, ^∗∗^*p* < 0.01 versus each treatment groups.

Consistent with the total ROS/RNS result, the activities of ROS-scavenging enzymes such as intracellular GPx and SOD levels were increased. Levels of myeloperoxidase activities in shungite-treated groups had decreased in serum and skin lysates, and this could be to compensate for other enzymes dissociating hydrogen peroxide (Figure 6).

### 3.4. Involvement of Nrf2 and p-ERK, p-p38 MAPK, and p-JNK Signaling Pathways in the Antioxidant Effect of Shungite against UVB-Induced Oxidative Stress

**Figure 7:**
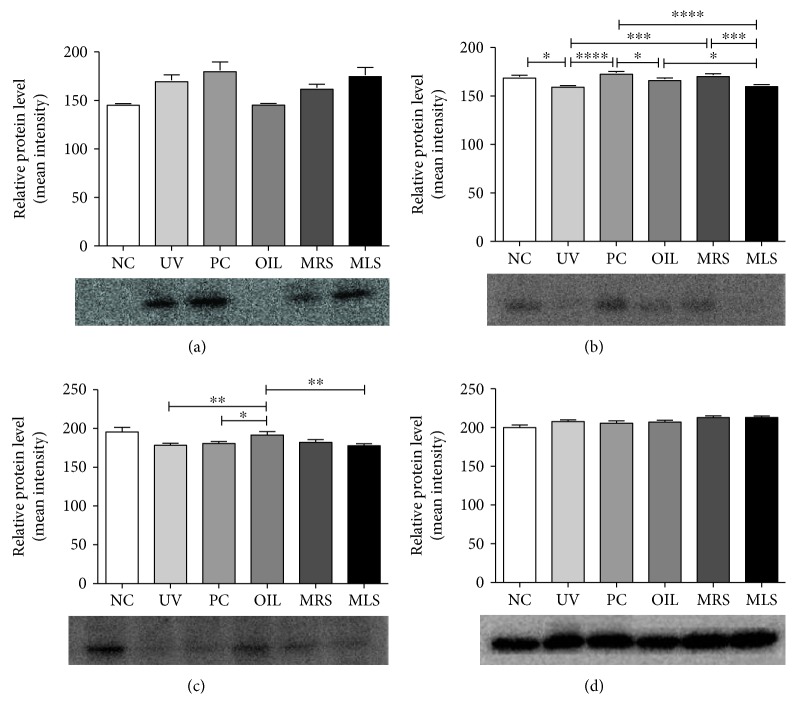
Nrf2 and MAPK proteins, P-ERK and p-JNK, are involved in the antioxidant effect of shungite against UVB-induced oxidative stress. Signaling cascade controlling cellular responses were measured for protein expression. Mineral-rich shungite (MRS) and mineral less shungite (MRS) induced phosphorylation of p-ERK (a), p-JNK (b) and Nrf2 (c), and *β*-actin (d) signaling pathway. Control groups include non-UVB-irradiated group (NC), UVB-irradiated only group (UV), UVB + positive control fullerene-treated group (PC), and UVB + olive oil-treated group (OIL), while experimental groups include UVB + mineral-rich shungite-treated group (MRS) and mineral-less shungite-treated group (MLS). Statistical analysis was carried out using analysis of variance (ANOVA) followed by subsequent multiple comparison test (Tukey's multiple comparisons test) using GraphPad Prism version 5.0. All values are represented as mean ± SD, *n* = 9, ^∗^*p* < 0.05, ^∗∗^*p* < 0.01, ^∗∗∗^*p* < 0.001, and ^∗∗∗∗^*p* < 0.0001 versus each treatment.

To fully elucidate the underlying molecular mechanism of antioxidant effect mediated by shungite, we hypothesized those different pathways that can lead to inflammatory. To prove our hypothesis, we determined whether the skin lysate of the shungite-treated group can induce the phosphorylation of Nrf2, p-p38 MAPK, and p-JNK.  Figure 7 showed that there was a marked increase in phosphorylation of Nrf2, p-p38 MAPK, and p-JNK. This result indicates that topical application of shungite can induce phosphorylation of Nrf2, p-p38 MAPK, and p-JNK. This study also found out that Nrf2 and MAPK proteins, p-ERK and p-JNK, are involved in the antioxidant effect of shungite against UVB-induced oxidative stress. Nrf2 is a transcription factor that regulates expression of many detoxification or antioxidant enzymes. It is plausible that fullerene transiently increases the intracellular level of ROS and/or activates p38 MAPK signaling pathway, which may possibly lead to facilitating the dissociation of Nrf2 from Keap. The resultant Nrf2/ARE activation induced phase II detoxification or antioxidant enzyme, thereby potentiating cellular defence capacity against cell death.

### 3.5. Conclusion

This study confirms the hypothesis that shungite, a natural fullerene, has antioxidant properties as it reduced the intracellular ROS production and enhanced the antioxidant enzyme activities (GPx, SOD, and MPO) *in vivo*. It is the first note to show several mechanisms and evidences of shungite's antioxidant effects including the improvement of several skin test parameters (moisture, elasticity, roughness, pore size, pigmentation, and wrinkle) and recovery of total WBC. Most importantly, shungite counterbalanced ROS/antioxidant paradigm as shown by the reduction of ROS/RNS levels and superoxide levels in both serum and skin lysate levels. Consistently, the activities of ROS-scavenging enzymes such as GPx and SOD levels were increased. In line, the inflammatory markers such as the cytokine levels of IL-1*β*, IL-6, IL-10, Il-17, IL-KC, and TNF-*α* in mice serum and skin lysates were lower than those in the NC group. Last, we found that Nrf2 and MAPK proteins, p-ERK and p-JNK, were involved in the antioxidant effect of shungite against UVB-induced oxidative stress. Given these, our data verify that mineral-rich shungite and mineral-less shungite were effective in the overall screening of its antioxidant and anti-inflammatory properties compared to the other control treatments. Further, MLS has a stronger antioxidant and anti-inflammatory effects than MRS. Synthesizing these, we rationally infer that shungite could be a one potential agent in such other diseases as well and can also be an alternative to the known applications of pure fullerene. However, the comparison of shungite-treated groups to other potent agents on other skin damage and immune- and oxidative-related diseases such as psoriasis or atopic dermatitis was not part of the study, but could be one of the future study targets. Since the processibility of pure fullerene is very challenging and very expensive, this study suggests that natural mineral shungite, as a novel antioxidant, could provide a new therapeutic insight against oxidative- and inflammatory-related diseases.

## Figures and Tables

**Table 1:** Slight suppression of total white blood cells (WBC) and their differential counts in the mineral-rich shungite-treated group. Shown are the effects of mineral-rich shungite (MRS) and mineral-less shungite (MLS) on the total WBC (a) and their differential counts including neutrophil (b), lymphocyte (c), monocyte (d), basophil (e), and eosinophils (f). Control groups include non-UVB-irradiated group (NC), UVB-irradiated only group (UV), UVB + fullerene C60-treated positive control group (PC), and UVB + olive oil-treated group (OIL), while experimental groups include UVB + mineral-rich shungite-treated group (MRS) and mineral-less shungite-treated group (MLS). Statistical analysis was carried out using analysis of variance (ANOVA) followed by subsequent multiple comparison test (Tukey's multiple comparisons test) using GraphPad Prism version 5.0. Data were expressed as mean ± SD, *n* = 9.

Units in K/mL	NC	UV	PC	OIL	MRS	MLS
Total WBC	3.16 ± 0.83	3.098 ± 0.91	4.25 ± 1.94	3.54 ± 2.22	3.032 ± 0.66	3.76 ± 1.26
Neutrophil	1.12 ± 0.35	1.326 ± 0.76	1.59 ± 0.86	1.31 ± 0.86	1.03 ± 0.28	1.41 ± 0.44
Lymphocyte	1.97 ± 0.62	2.094 ± 0.63	2.34 ± 0.96	1.99 ± 1.41	1.82 ± 0.42	2.13 ± 0.82
Monocyte	0.13 ± 0.071	0.15 ± 0.90	0.22 ± 0.11	0.18 ± 0.13	0.13 ± 0.04	0.17 ± 0.07
Eosinophil	0.02 ± 0.013	0.041 ± 0.058	0.076 ± 0.13	0.05 ± 0.06	0.034 ± 0.084	0.033 ± 0.044
Basophil	0.007 ± 0.0048	0.011 ± 0.017	0.027 ± 0.048	0.014 ± 0.017	0.019 ± 0.036	0.013 ± 0.016
